# Adenosine at the Interphase of Hypoxia and Inflammation in Lung Injury

**DOI:** 10.3389/fimmu.2020.604944

**Published:** 2021-01-14

**Authors:** Xiangyun Li, Nathanial K. Berg, Tingting Mills, Kaiying Zhang, Holger K. Eltzschig, Xiaoyi Yuan

**Affiliations:** ^1^ Department of Anesthesiology, McGovern Medical School, University of Texas Health Science Center at Houston, Houston, TX, United States; ^2^ Department of Anesthesiology, Tianjin Medical University NanKai Hospital, Tianjin, China; ^3^ Department of Biochemistry, McGovern Medical School, University of Texas Health Science Center at Houston, Houston, TX, United States

**Keywords:** adenosine, inflammation, hypoxia, hypoxia-inducible factor, acute lung injury, chronic lung injury

## Abstract

Hypoxia and inflammation often coincide in pathogenic conditions such as acute respiratory distress syndrome (ARDS) and chronic lung diseases, which are significant contributors to morbidity and mortality for the general population. For example, the recent global outbreak of Coronavirus disease 2019 (COVID-19) has placed viral infection-induced ARDS under the spotlight. Moreover, chronic lung disease ranks the third leading cause of death in the United States. Hypoxia signaling plays a diverse role in both acute and chronic lung inflammation, which could partially be explained by the divergent function of downstream target pathways such as adenosine signaling. Particularly, hypoxia signaling activates adenosine signaling to inhibit the inflammatory response in ARDS, while in chronic lung diseases, it promotes inflammation and tissue injury. In this review, we discuss the role of adenosine at the interphase of hypoxia and inflammation in ARDS and chronic lung diseases, as well as the current strategy for therapeutic targeting of the adenosine signaling pathway.

## Introduction

Acute respiratory distress syndrome (ARDS) is common in critically ill patients, characterized by respiratory failure, pulmonary edema independent of left heart failure, as well as high morbidity and mortality (1). The mortality rate was 30–40% in the most recent studies despite the latest improvement in clinical management (2). Pathological characters of ARDS in the acute “exudative” phase (~7 days) include alveolar epithelial and endothelial injury, resulting in interstitial and alveolar edema, hyaline membrane formation, and alveolar hemorrhage, as well as the accumulation of immune cells (1, 3). The main causes for ARDS include pneumonia, aspiration of gastric contents, severe trauma as well as sepsis (1, 4, 5). The recent global outbreak of Coronavirus disease 2019 (COVID-19) has placed viral infection-induced ARDS under the spotlight. COVID-19 is caused by severe acute respiratory syndrome coronavirus 2 (SARS-CoV-2) infection and has resulted in a worldwide pandemic rapidly because of its high transmissibility and pathogenicity (6). ARDS is one of the most common organ dysfunctions for severe COVID-19, which accounts for the cause of death in 70% of fatal cases (7, 8). There are several emerging viruses in the past 20 years, which can induce ARDS-related mortality, such as influenza H1N1 2009, influenza H5N1 and H7N9 viruses, the severe acute respiratory syndrome coronavirus (SARS), and Middle East respiratory syndrome coronavirus (MERS) (9). It is reported that about 30–40% of the hospitalized patients infected with influenza virus progress to pneumonia, and influenza A shows a higher predisposition to ARDS in adults (10). Compared to SARS (10%) and MERS (35%), COVID-19 shows lower mortality rates of approximately 5.2%, but higher infectiousness (9, 11). As of September 6^th,^ 2020, the pandemic of COVID-19 had affected over 26 million individuals around the world and caused more than 800,000 deaths worldwide. Therefore, the search for effective therapeutic approaches for the preventing and treatment of COVID-19 associated ARDS has become an urgency. Currently, although there are certain improvements in the management of ARDS, the treatment for ARDS is in urgent need. Therefore, the fundamental pathogenesis and effective treatments for ARDS are still under intensive investigation.

Persistent pulmonary inflammation and tissue remodeling result in the gradual decline in pulmonary function in patients suffering from chronic lung diseases including chronic obstructive pulmonary disease (COPD), asthma, and idiopathic pulmonary fibrosis (IPF) (3, 12–16). Chronic lung disease ranks the third leading cause of death in the United States. The risk factors of chronic lung diseases included genes, environmental factors, and aging (3, 12, 13, 17). However, one of the common characteristics among these diseases is dysregulated recruitment or activation of immune cells, such as neutrophils, macrophages, dendritic cells, and other effector cells, such as fibroblasts, myofibroblasts, and airway epithelial cells (AECs), which accelerates pulmonary remodeling and inflammatory response (3, 4). The therapeutic approaches for chronic lung diseases focus on providing symptomatic relief, but pharmacologic compounds are still lacking to reverse the profound tissue remodeling and restore lung function in these patients.

Adenosine was first isolated from the heart muscle and identified as an “adenine compound” that could change cardiac rhythm when injected in guinea pigs in 1927 (18). Besides its function in cardiac rhythm, adenosine also modulates inflammatory responses during hypoxic conditions (19–21). In this review, we will discuss the interaction between hypoxia and adenosine signaling, including adenosine, adenosine receptors, and adenosine metabolism, in acute lung inflammation and chronic lung diseases. We will also focus on the currently available approaches for therapeutic targeting of the hypoxia-adenosine axis in these disease conditions.

## Biology of Adenosine

### Extracellular Adenosine Generation

**Figure 1:**
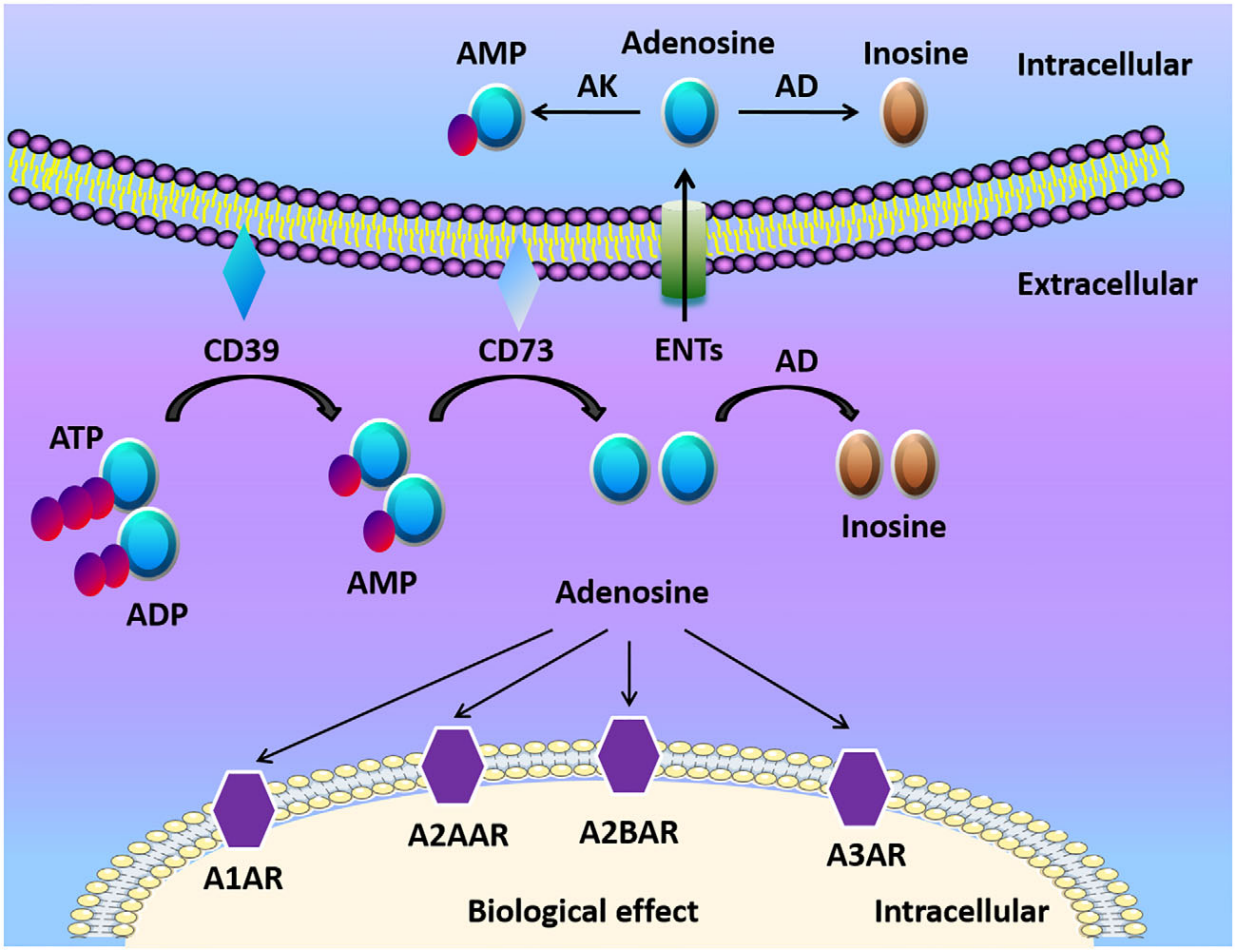
Adenosine biogenesis and signaling. ATP and ADP are the main resources of extracellular adenosine. ATP and ADP are dephosphorylated to AMP on the cell surface by Ecto-nucleotide triphosphate diphosphohydrolase 1 (CD39) and ecto-5’-nucleotidase (CD73) dephosphorylates AMP to adenosine. Adenosine activates adenosine receptors (A1AR, A2AAR, A2BAR, A3AR) and plays a crucial role in different cells and organs. Adenosine can be transported into the cell by equilibrative nucleoside transports (ENTs), or be transformed to inosine *via* CD26-bound adenosine deaminase (ADA) at the cell surface. Under normoxic conditions, adenosine has a high affinity with adenosine receptors and ENTs. Under hypoxia conditions, the release of extracellular ATP/ADP increased. Finally, HIFs enhanced the release of extracellular adenosine and adenosine receptors, which modulates tissue barriers and inflammatory response.

Adenosine, along with ATP and ADP, is considered the main purinergic signaling molecules (**Figure 1**). The release of ATP from intracellular to the extracellular environment contributes to the formation of adenosine especially when the tissue is in inflammatory, ischemic, and hypoxic conditions (23, 24). ATP/ADP in the extracellular space can be converted to adenosine monophosphate (AMP) by ectonucleoside triphosphate diphosphohydrolase-1 (CD39) (25, 26). Then AMP is further converted by ecto-5’-nucleotidase (CD73) to extracellular adenosine (25, 26). Mice with CD39 or CD73 deficiency are viable, which indicates that nucleotide phosphohydrolysis regulated by ectoenzymes is not vital in regular physiologic conditions (27). However, ectonucleotidases still have a crucial role in disease conditions. For example, the upregulation of adenosine generation and CD39 and CD73 expression is one of the protective mechanisms to reduce apoptosis, and alleviate inflammation in kidney ischemia/reperfusion (I/R) injury models (28). The deletion of CD39 in mice leads to increased level of ATP/ADP, and reduced adenosine levels, along with elevated risk of dysregulated inflammation and tissue injury (29, 30). Similarly, genetic deletion of CD73 results in higher mortality and delayed acute lung injury resolution when compared with WT mice because of the dampened generation of adenosine in regulatory T cells (Tregs) (31). Therefore, the conversion of ATP/ADP to adenosine is considered beneficial in many ischemic and inflammatory disorders.

### Adenosine Receptors and Signaling

Adenosine receptors, which include four distinct G-protein coupled seven membrane-spanning cell surface receptors: the adenosine A_1_ receptor (A1AR), the adenosine A_2A_ receptor (A2AAR), the adenosine A_2B_ receptor (A2BAR), and the adenosine A_3_ receptor (A3AR), are crucial for adenosine mediated responses (3, 19, 21, 27). Both A2AAR and A2BAR are linked to Gs protein involving activation of adenylate cyclase, to stimulate cAMP production followed by PKA activation (32–35). A1AR and A3AR, on the other hand, bear a distinct signal transduction pathway. For example, A1AR activation inhibits cAMP accumulations in Chinese hamster ovary cells (36). The coupling of A1AR to the Gi/o protein pathway attenuates cAMP signal transduction in hepatic stellate cells (33). Furthermore, A3AR has been indicated to attenuate adenosine-induced increase of cAMP in rat vascular smooth muscle cells *in vitro* (37) and A3AR knockout mice show an increased level of cAMP in the cardiovascular system (38). Functionally, Dr. Michail Sitkovsky’s laboratory identified that A2AAR is crucial for limiting inflammatory responses as mice with A2AAR deficiency showed profound tissue damage in inflammation and endotoxin-induced septic shock (21). The expression of adenosine receptor subtypes is different in various cell types. For example, neutrophils and lymphocytes have higher expression levels of A2AAR, while vascular endothelial cells have higher levels of A2BAR (39–41). It has been elucidated that adenosine receptors have important functions in pathologic conditions. For instance, adenosine has a selective role in reducing the heart rate *via* A1AR, which would be a potential therapeutic method for superventricular tachycardia in mice (42). Adenosine signaling *via* A2AAR or A2BAR has a beneficial effect *via* shifting proinflammatory immune response to anti-inflammatory immune response as well as promoting barrier protection in different animal models (43–48). A3AR is related to the aqueous humor production in the eye in a preclinical study (49), and its agonist showed efficacy in treating dry eye syndrome in a clinical study (50).

### Intracellular Adenosine Metabolism

The termination of adenosine signaling is mediated by the transportation of adenosine from the extracellular to the intracellular space (**Figure 1**) (27, 51). ENTs and concentrative nucleoside transporters (CNTs) are nucleoside transporters found on various cell types (52, 53). According to the concentration gradient, adenosine moves freely across these channels because of its diffusion-limited character (53). Adenosine signaling can be diminished by the transportation of adenosine into the cell and then metabolized to inosine *via* adenosine deaminase (ADA) (54). Additionally, adenosine kinase can convert adenosine to AMP (55). The activation of mucosal A2B signaling combined with the repression or deletion of epithelial ENT2 dampens mucosal inflammation (56). Another study also showed that elevations of adenosine protect from liver injury after the genetic deletion or inhibition of Ent1 *via* A2B signaling in liver ischemia and reperfusion models (57).

## Hypoxia and Inflammation in Lung Injury

**Figure 2:**
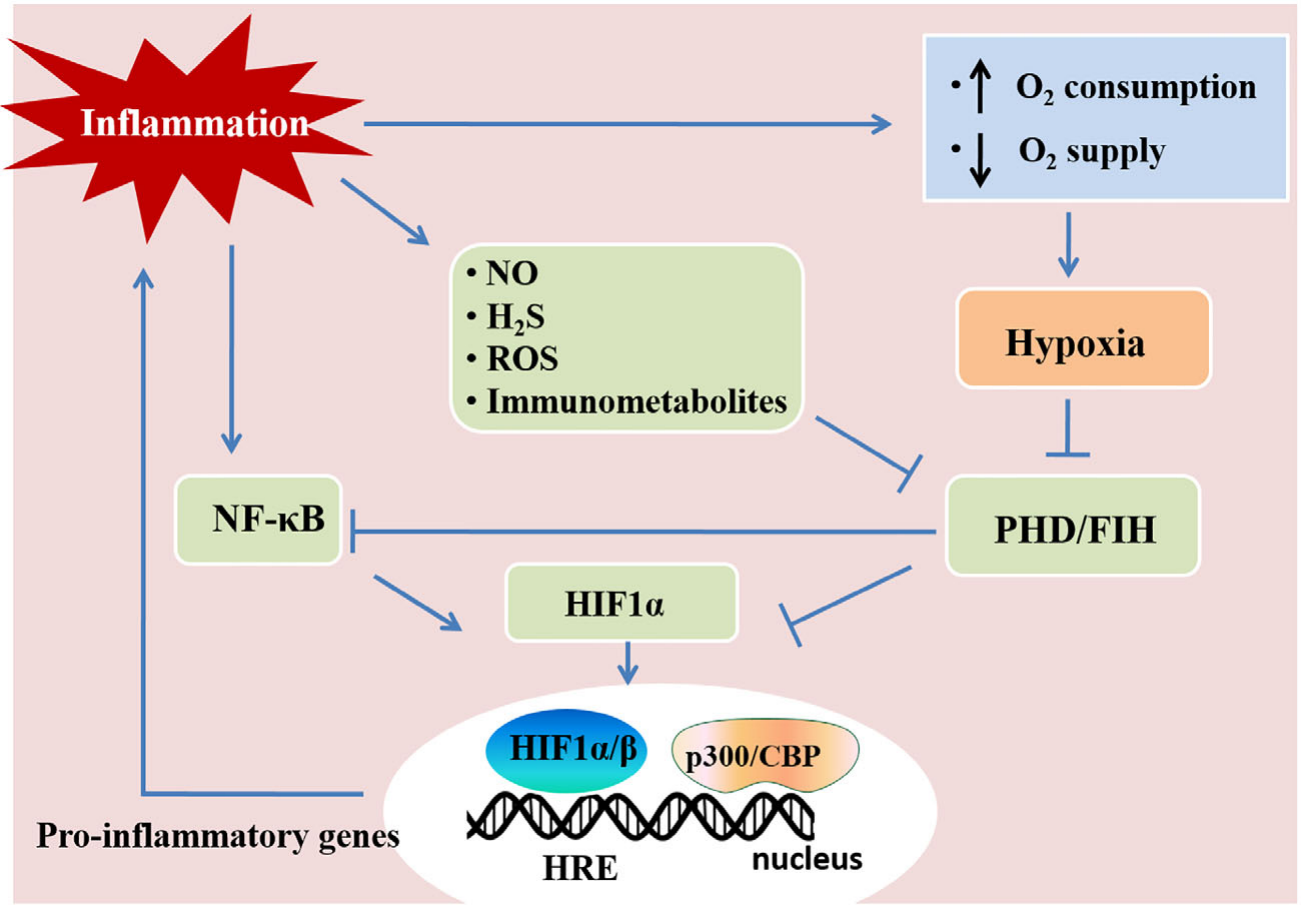
Hypoxia and inflammation. Inflammation and hypoxia are co-incidental events in several pathological conditions. Inflammatory stimuli, such as cytokines, bacterial products, and hypoxia, activate the nuclear factor-κB (NF-κB) pathway. The activation of NF-κB enhances the transcription of HIF-1α mRNA and promotes HIF activity. Inflammatory mediators, such as nitric oxide (NO), hydrogen sulfide (H_2_S), reactive oxygen species (ROS), and immunometabolites also control HIF activity in immune cells, which regulates immunity and inflammation. Activated HIF-1α translocates to the nucleus and promotes the transcription of pro-inflammatory genes by associating with HIF-1β and the cofactor p300/CBP. This figure is adapted from Regulation of immunity and inflammation by hypoxia in immunological niches; Cormac T. Taylor and Sean P. Colgan, *Nature Reviews Immunology;* 17, pages 774–785(2017) (22).

Hypoxia and inflammation frequently occur in pathogenic conditions such as cancer, inflammatory bowel diseases, ischemia/reperfusion injury, and inflammatory lung diseases (58). Hypoxia-inducible factors (HIFs) are crucial in the responses mediating the crosstalk between hypoxia and inflammation. Hypoxia-inducible factors (HIFs) have a central role in regulating tissue adaptation to low oxygen conditions. HIFs belongs to αβ-heterodimeric transcription factors that include HIF-1α, HIF-2α, and HIF-1β/ARNT subunits. When oxygen is abundant, HIF-1α or HIF-2α binds to the von Hippel-Lindau (VHL) gene product, a part of the E3 ubiquitin ligase complex, and result in proteasomal degradation (59–61). HIFα and VHL binding are related to the hydroxylation of HIFα proline residues, which rely on prolyl hydroxylases (PHDs) and factor-inhibiting HIF (FIH) (60, 61). Under hypoxia, HIFα subunits can not be hydroxylated as efficiently due to the lack of oxygen as a substrate for PHDs, which results in the stabilization of HIF-1α and HIF-2α. Once stabilized, HIFα translocates to the nucleus and binds to HIF-1β to form a complex, and in turn bind to hypoxia-responsive elements (HRE) of the promoter region in the target genes for start transcriptional regulation (46, 62, 63). Most of the HIFs target genes are related to metabolism, proliferation, oxygen transport, and other processes important for hypoxia adaptation (64). HIF stabilization is demonstrated in inflammatory conditions and diseases, such as lung injury, inflammatory bowel disease, and ischemia-reperfusion injury through various mechanisms (**Figure 2**) (3, 58, 65). Tissue metabolism in inflammatory disease has higher local oxygen demand, which induces tissue hypoxia. Additionally, the supply of oxygen decreased due to the shortage of tissue blood supply in trauma, ischemia, and vascular occlusion disease, which aggravate tissue inflammation (46, 66). Moreover, cytokines (e.g., IL-1, IFN-β, TNF-α) released during inflammation have an impact on HIF-1α expression (67, 68). The hypoxic environment in solid tumors and during ischemia/reperfusion activates NF-κB, which is a crucial transcription factor regulating inflammation and immune response (69–71). Therefore, hypoxia and inflammation usually occur simultaneously during pathogenic conditions, and they are closely linked to each other.

### Acute Respiratory Distress Syndrome

Recently, increasing research effort has provided convincing evidence of the link between hypoxia and inflammation in ARDS (72–75). For example, HIF-1α is stabilized under normoxic conditions by mechanical stretch of alveolar epithelial cells *in vitro* and in ventilation-induced lung injury (VILI) in mice *in vivo* (72). The normoxic stabilization of HIF-1a by mechanical stretch could be explained by the inhibition of succinate dehydrogenase (SDH). Functionally, HIF-1α stabilization dampens lung inflammation through the regulation of glucose metabolism in alveolar epithelial cells, because only mice with alveolar epithelial cell-specific deletion of HIF-1α show profoundly increased lung inflammation and pulmonary edema (72). The protective effect of HIF-1α in alveolar epithelial cells has also been demonstrated in acute cobalt-induced lung injury models as more neutrophilic infiltration and Th1 cytokines were observed in alveolar epithelial-specific HIF-1α-deficient mice (76). Additionally, HIF-2α activation improved endothelial adherens junction integrity in endotoxin-mediated injury through increasing its target gene vascular endothelial protein tyrosine phosphatase (VE-PTP) (77). Furthermore, the pharmacologic activator of HIF, dimethyloxalylglycine (DMOG), protects the lung alveolar epithelium during murine VILI and LPS induced acute lung injury *via* enhancement of glycolysis (72, 78). Another study showed that DMOG treatment attenuates Fas Ligand (FasL)-induced apoptosis in MLE-12 cells *in vitro* and dampens lung inflammation, and histopathological changes intratracheal FasL induced lung injury in mice *in vivo* (79). These studies suggests that pharmacological HIF activator could offer lung protection during ARDS *via* maintaining alveolar epithelial and endothelial functions during injury.

Viral infection-induced ARDS has been the center of attention because of the recent pandemic of COVID-19. Influenza virus infection is one of the most studied models for viral pneumonia (80–83). Several studies have shown a close relationship between hypoxia and inflammation in viral infection-induced ARDS. For example, respiratory syncytial virus infection in mice results in the stabilization of HIF-1α in an oxygen-independent manner (84). Besides, earlier studies indicated that influenza A (H1N1) virus infection could induce HIF-1α nuclear translocation but did not change its expression levels in A549 cells *in vitro* (85). A recent study indicated that H1N1 infection stabilizes HIF-1α under normoxic conditions in A549 cells *in vitro* and in murine models of H1N1 mediated viral pneumonia *in vivo* (86). The normoxic stabilization of HIF-1α is dependent on the inhibition of proteasome function and decreasing the expression of factor inhibiting HIF-1 (FIH-1) (86). Moreover, influenza A virus (IAV) infection-induced acute lung injury (ALI) also results in hypoxia, and further contribute to the stabilization of HIF-1α in mouse lung tissue (87). Functionally, alveolar epithelial type II cell-specific deficient *Hif1a^fl/fl^* SPCCre mice showed increased lung inflammation and mortality during IAV infection *in vivo* (87). Mechanistically, HIF-1α deficiency promotes influenza A virus replication in A549 cells *in vitro via* reducing glycolysis and enhancing autophagy (87). The functional role of HIF in SARS-CoV-2 infection associated ARDS needs to be further investigated.

### Chronic Lung Injury

IPF is one of the most common and severe forms of interstitial lung disease (88). IPF patients suffer from an impaired pulmonary gas exchange and chronic arterial hypoxemia (89). The important role of hypoxia and HIFs on fibroblast proliferation and differentiation has been studied extensively (90–92). Besides the direct impact on fibroblasts, hypoxia is regarded as one of the potent stimuli for the production of proinflammatory cytokines. For example, protein kinase C (PKC) activation promotes the expression of TNF-α and IL-1β in the pulmonary artery under hypoxic conditions (93). Additionally, vascular endothelial growth factor (VEGF), a known target gene of HIF, is an angiogenesis factor with proinflammatory, permeability-inducing roles in murine bleomycin-induced pulmonary fibrosis (94). Furthermore, HIF-1α stabilization has been observed in alternatively activated macrophages in a murine model of bleomycin-induced pulmonary fibrosis and HIF-1α inhibition in macrophages inhibits the expression of profibrotic mediators including IL-7 and CXCL1 (95). However, the involvement of hypoxia signaling in other subtypes of immune cells during IPF has yet to be elucidated.

Inflammation and hypoxia are also tightly linked in COPD, including chronic bronchitis and emphysema. For example, cigarette smoking significantly increases inflammation mediators expression, such as IL-6, IL-8, and TNF-α (96). These factors contribute to the activation of hypoxia response genes (including HIFs, NF-κB) and promote the development of COPD in rats (97). HIFs are overexpressed in the lung tissue of COPD patients (98) and HIF-1α level is positively correlated with the severity of COPD in patients (99). HIF-2α, on the other hand, has been shown to be decreased in lung tissue from emphysema patients compared to healthy control (100). Furthermore, endothelial cell-specific deletion of HIF-2α in mice results in emphysematous changes in the lung, which was exaggerated by the treatment of SU5416, a vascular endothelial growth factor receptor 2 (VEGFR2) inhibitor (100). On the other hand, endothelial-specific overexpression of HIF-2α in mice was protected from emphysema (100), suggesting therapeutic activation of HIF-2 α as a treatment for emphysema.

Hypoxia is frequently encountered in patients suffering from severe asthma or acute exacerbation (101). How hypoxia and HIFs influence allergic airway inflammation has been studied extensively. An earlier study suggested that HIF-1α is stabilized in lung tissues from asthmatic patients and in a murine model of allergic airway inflammation induced by ovalbumin sensitization (102). This study also demonstrated that deficiency in HIF-1β significantly dampens allergic airway inflammation and reduced ovalbumin-specific antibodies in mice (102). Consistently, HIF-1α antagonist YC-1 reduced airway hyperresponsiveness and lung inflammation in a murine model of asthma (103, 104). Besides its global impact on allergic airway inflammation, the functional role of HIF-1α in different subsets of immune cells has also been investigated in asthma. For instance, myeloid-specific deletion of HIF-1α in mice results in reduced airway hyperresponsiveness (AHR), and HIF-1α deficient eosinophils show reduced chemotaxis (104). Furthermore, a recent study indicates that exposure to 3% oxygen leads to increased T helper type 2 cells (Th2) cytokine expression in CD8^+^ T cells and adoptive transfer of these cells exaggerated AHR and lung inflammation in the ovalbumin model of murine allergic airway disease (105). Additionally, HIF-1α inhibition reduced Th2 cytokines expression in CD8^+^ T cells upon hypoxia exposure, and the adoptive transfer of HIF-1α deficient CD8^+^ T cells underwent hypoxia attenuates AHR and airway inflammation in mice (105). In summary, HIF-1α is important for the development of AHR and airway inflammation by modulating immune cell chemotaxis and function. However, the detailed mechanism, such as the identification of HIF target genes in specific immune cells during asthma, needs to be further investigated.

## Adenosine at The Interphase of Hypoxia and Inflammation in Lung Injury

**Figure 3:**
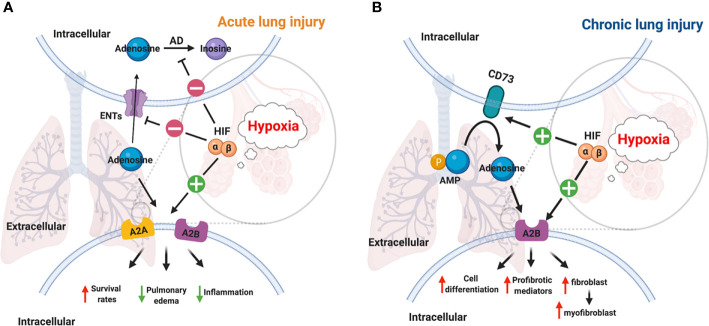
Links between HIF and adenosine signaling in acute/chronic lung injury. **(A)** Inflammation and infection results in the stabilization of HIFs in acute lung injury (112). HIF-1a dependent inhibition of ENT1, ENT2, and adenosine kinase contributes to the accumulation of adenosine (55, 123). A2AAR and A2BAR are two adenosine receptors that are regulated by HIF-2α and HIF-1α respectively in lung tissue (109, 120, 121). Therefore, the higher level of adenosine, and the activation of its receptors reduced mortality, pulmonary edema, inflammation in acute lung injury. **(B)** The activation of the hypoxic-adenosinergic system has been investigated in chronic lung injury. CD73 and A2BAR are two hypoxia-inducible genes in patients with idiopathic pulmonary fibrosis and pulmonary hypertension (124). The upregulation of A2BAR enhances cell differentiation, produces profibrotic mediators, and promotes fibroblast to myofibroblast in chronic lung injury (95, 125).

In the past decades, studies have provided ample evidence that hypoxia signaling is tightly linked with adenosine signaling (46, 58, 106–112). Previous studies showed that hypoxic condition or inflammation contributes to the accumulation of extracellular ATP/ADP due to the damage in the cell membrane (3, 23, 27, 113–115). The increased level of extracellular ATP and ADP is essential for the generation of extracellular adenosine, which is a key mediator of inflammatory responses (116, 117). It has been demonstrated that HRE in the promoter of CD73 gene is crucial for HIF-1α mediated expression in epithelial cells under hypoxic conditions, and the inhibition of HIF-1α decreases the hypoxia-inducible CD73 expression (118). Besides HIF-1α, transcription factor Sp1 is also involved in the transcription of CD39 under hypoxia conditions, and its protective effect has been demonstrated during cardiac and hepatic ischemia (29, 46, 119). Moreover, A2AAR has been identified as a target gene of HIF-2α in human lung endothelial cells (120), while A2BAR has been identified as a target gene of HIF-1α (121, 122). The links between HIF and adenosine are not only through regulation of ectonucleotidases and adenosine receptors but also *via* equilibrative nucleoside transporters (ENTs) and its G-protein-coupled receptors. For example, HIFs are implicated in the repression of ENT1 and ENT2 (53, 123) and abolish the conversion of adenosine to AMP by adenosine kinase in cells (46, 55). The close relationship between hypoxia and adenosine signaling in acute and chronic lung injury have been established during the past decades (**Figure 3**).

### Extracellular Adenosine Generation

Several studies suggest that adenosine level increases following hypoxia exposure in animal studies (126) and in human studies (127, 128). *In vitro* cell culture experiments and *in vivo* animal studies indicated that endogenous adenosine generation inhibits neutrophil accumulation during hypoxia (129). Particularly, CD39 deficient mice show an increased level of MPO in colon, lung, kidney, and liver after 4 h of exposure to hypoxia (8% O2) compared to wild-type mice. Pharmacological inhibition or genetic deletion of CD73 in mice leads to a similar phenotype as CD39 deficient mice, suggesting the importance of extracellular adenosine generation in hypoxia-induced inflammation. Moreover, short term exposure to hypoxia increases plasma levels of adenosine, attenuates pro-inflammatory cytokine release, and results in an elevated level of IL-10 during experimental endotoxemia models in humans (130). Extracellular adenosine levels increase after the mechanical ventilation in mice or stretched pulmonary epithelial cells (106, 112). Pharmacological inhibition or genetic deletion of CD39 or CD73 in mice leads to severe lung inflammation with mechanical ventilation, suggesting the protective effect of adenosine (106). The relationship between HIF and adenosine in ARDS during viral pneumonia has not been clearly demonstrated yet. Nucleotide ATP and adenosine in BALF have been shown to be increased after the infection of influenza A virus in mice (131, 132). However, adenosine levels and pathogenesis of ALI did not show any difference between WT and CD73-knockout mice after the infection of influenza A virus. Therefore, CD73 is not considered as one of the crucial factors for the development of influenza-induced ALI (133).

Cellular stress and damage induce the generation of adenosine in lung tissue of patients with chronic lung disease. For example, the hypoxic-adenosinergic pathway is activated in IPF patients with pulmonary hypertension (PH), as marked by increased expression of HIF-1α, adenosine, adenosine A2B receptor, CD73, and ENT1 (124). Other studies showed that adenosine levels are increased in the serum, lymphocytes, and erythrocytes in healthy smokers compared to healthy non-smokers and continue to increase with the severity in COPD patients (134). The same study also demonstrated that patients with higher levels of adenosine tend to have reduced forced expiratory volume in one second (FEV1), suggesting a potential functional link (134). Furthermore, adenosine signaling is significantly enhanced in COPD as represented by increased CD73 activity and adenosine receptor levels in lung tissue from patients with COPD or in murine model of emphysema (135, 136). Adenosine signaling is also enhanced in asthma, and consequently, a high level of adenosine induces airway hyperresponsiveness and bronchoconstriction and promotes human mast cells to release allergen-induced mediators (137).

### Adenosine Receptors and Signaling

#### Adenosine A_1_ Receptor

A1AR has diverse roles in lung injury. For example, A1AR deficient mice have increased susceptibility to LPS-induced acute lung injury with increased PMN recruitment and microvascular permeability (138). The same study indicated that pretreatment of A1AR agonist, 2’Me-2-chloro-N6-cyclopentyladenosine, attenuates PMN recruitment and microvascular permeability. On the other hand, post-infection treatment of a combination of A1AR antagonist L-97-1 and ciprofloxacin improves the outcome of *Y. pestis* infection in rats, indicating a protective effect of A1AR (139). Furthermore, A1AR knockout mice show significantly increased macrophage and neutrophil infiltration in the airway after influenza A/WSN/33 (H1N1) infection when compared to wild-type counterparts and daily treatment of A1AR antagonist 8-cyclopentyl-1,3-dipropylxanthine results in improved outcome (140). Besides acute lung injury and infection, the activation of A1AR has also been found on bronchial epithelial cells, and inflammatory cells, which enhanced the asthma phenotype (141). Mice with ADA deficiency experience lung injury and inflammation (142). A1AR deletion in mice exaggerated the pulmonary inflammation marked by increased expression of IL-4 and IL-13, as well as matrix metalloproteinases, suggesting a protective role of A1AR in chronic lung injury (142).

#### Adenosine A2A Receptor

Exposure to hypoxia (10% O_2_) attenuates lung inflammation during LPS-induced lung injury in mice and A2AAR is indispensable for hypoxia-mediated lung protection (143). A2AAR expression in myeloid cells is crucial for the control of neutrophil recruitment to the lung injury and an A2AAR specific agonist ATL202 offers lung protection in mice during LPS-induced lung injury (144). The lung protective effect of A2AAR has also been implicated in cardiopulmonary bypass-mediated lung injury. Pretreatment of A2AAR agonist CGS21680 in juvenile rats dampens inflammatory cytokines and myeloperioxidase levels in the serum as well as pulmonary edema and lung injury score during cardiopulmonary bypass−induced organ injury. Another study demonstrated that A2AAR activation induces the expression of peroxisome proliferator-activated receptors γ (PPARγ) *via* cAMP and PKA pathways in murine macrophages (145). Combining PPARγ agonist ROSI and A2AAR agonist CGS21680 significantly reduces lung pathology and the production of inflammatory cytokines in the lung during murine model of LPS-induced ALI (145). Moreover, treatment of CGS21680 after the onset of trauma/hemorrhagic shock-induced lung injury attenuates pulmonary edema and MPO levels in Sprague-Dawley rats (146). Interestingly, A2AAR has been identified as a HIF-2α target in pulmonary endothelial cells *in vitro*, implicating the crosstalk between adenosine signaling and hypoxia signaling (120). Furthermore, treatment of A2AAR agonist CGS21680 reduces inflammatory cell infiltration to the airway in murine models of asthma (147). A2AAR deficient mice experience exaggerated lung inflammation and airway hyperactivity, suggesting the protective role of A2AAR in allergic airway diseases.

#### Adenosine A2B Receptor

A2BAR is an important link between hypoxia and adenosine signaling in acute lung injury. HIF-1α has been shown to transcriptionally induce the expression of A2BAR in murine VILI model (109, 121). For instance, genetic silence or pharmaceutical inhibition of HIF-1α dampens the expression of A2BAR in mice during VILI or alveolar epithelial cells exposed to cyclic stretch (109). A2BAR-dependent adenosine signaling offers lung protection during endotoxin-induced ALI in mice by potentiating the regulatory T cell population (148). Furthermore, hypoxia-induced vascular leakage also exaggerates in siRNA-mediated knockdown of A2BAR or A2BAR deficient mice (149). Furthermore, HIF-1α-dependent induction of netrin-1 attenuated neutrophil transmigration and dampens inflammation through A2BAR at pulmonary and colon mucosal surface (150), suggesting another layer of complexity in the crosstalk between HIF and adenosine signaling.

In chronic lung injury, hypoxia potentiates the function of adenosine and promotes the production of IL-6, and induce the differentiation of fibroblasts to myofibroblasts by increasing adenosine A2B receptor expression in human fibroblasts (125). Furthermore, adenosine deaminase-deficient mice have higher expression of A2BAR and exhibit progressive pulmonary fibrosis and respiratory distress (151). The crosstalk between hypoxia and adenosine signaling has been established in the murine model of IPF (95). For example, HIF-1α inhibition *via* the treatment of 17-DMAG results in reduced pulmonary fibrosis and A2BAR expression in the late stages of murine bleomycin-induced lung fibrosis *in vivo* (95). Additionally, HIF-1α inhibition along with A2BAR deletion or pharmacological inhibition result in disruption of alternatively activated macrophages differentiation and IL-6 production *in vitro* (95).

Of note, while A2AAR and A2BAR reduce mortality, pulmonary edema, and inflammation in acute lung injury, A2BAR enhances cell differentiation, produces profibrotic mediators, and promotes fibroblast to myofibroblast differentiation in chronic lung injury. The differential role of A2BAR could possibly be stemming from the different impacts of downstream signaling in acute or chronic lung injury. As mentioned above, A2AAR and A2BAR activation lead to the activation of cAMP and PKA pathway (32–35). The cAMP-CREB axis is important for the maintenance of endothelial integrity and the attenuation of lung inflammation during endotoxin-induced lung injury in mice (152). The protective effect of cAMP in LPS-induced endothelial permeability is mediated through PKA (153). cAMP synthesis and PKA activity are inhibited in oleic acid-induced lung injury, and the treatment of hydroxysafflor yellow A enhances the cAMP/PKA pathway and dampened lung inflammation in mice (154). Furthermore, pretreatment of phosphodiesterase antagonist PTX enhances cAMP signaling and results in the attenuation of lung injury during cecal ligation and puncture in mice (155). These studies suggest a protective role of cAMP and PKA during acute lung injury. In chronic lung injury, cAMP and PKA regulate hypercontractility in human airway smooth muscle cells (156) and phosphodiesterase inhibitors, which prevents the breakdown of cAMP, are currently being studied as a treatment for asthma (157). In addition, dibutyryl-cAMP treatment increases endogenous cAMP levels, enhances PKA signaling *in vitro*, and blocked myofibroblast differentiation *in vivo* (158). Other cAMP elevating agents also inhibits the proliferation and collagen production in pulmonary fibroblasts (159). Thus, the divergent function of A2BAR in acute and chronic lung injury might not be based on the downstream activation of the cAMP and PKA signaling pathway. Other factors could contribute to the response to cAMP activation as lung fibroblasts from pulmonary fibrosis patients has a deficiency in the phosphorylation of cAMP response element-binding protein (160). Future studies are needed to elucidate the signaling mechanism of A2BAR mediated responses in pulmonary injuries.

#### Adenosine A3 Receptor

A3AR is also expressed in the lung and several previous studies have indicated the functional role of A3AR in lung injury. The protective role of A3AR in lung ischemia/reperfusion injury has been demonstrated by an early study in which the pretreatment of A3AR agonist IB-MECA attenuated alveolar injury and apoptosis during lung ischemia and reperfusion injury of isolated cat lung *ex vivo* (161). The protective role of A3AR is further supported as pretreatment of IB-MECA offers lung protection during lung ischemia/reperfusion injury in cat *in vivo* (162). In addition, A3AR agonist CI-IB-MECA pretreatment alleviates lung ischemia/reperfusion injury in mice, and the protective effect is abolished in mice with genetic deletion of A3AR (163). Besides lung ischemia/reperfusion injury, the protective role of A3AR has also been indicated in LPS-induced lung injury. Indeed, A3AR deficient mice showed exaggerated PMN infiltration after LPS inhalation and pretreatment of CI-IB-MECA attenuates the inflammatory responses and injury (164). Furthermore, A3AR activation is associated with mast cell degranulation and airway hyperreactivity. For example, selective activation of A3AR *via* IB-MECA results in the release of histamine in mast cells *in vitro* and nebulizer treatment of IB-MECA in mice results in mast cell degranulation in the lung in wild type mice but not in A3AR deficient mice (165). Adenosine administration results in airway responsiveness in mice and mice with A3AR deficiency show attenuated responses marked by reduced mast cell degranulation and neutrophil infiltration (166). Other studies also demonstrate the contribution of A3AR in chronic airway inflammation (167, 168).

### Adenosine Metabolism

Besides the impact on adenosine receptors, HIF-1α dependent repression of ENT1 and ENT2 decreases adenosine uptake and increases extracellular adenosine, which dampens neutrophil accumulation and protects vascular barrier during hypoxia in endothelia and epithelia (123). HIF-1α-dependent repression of adenosine kinase leading to increased extracellular adenosine attenuates hypoxia-induced vascular leak in murine models of sepsis or ALI (169). In addition, adenosine deaminase activity, ADA2 in particular, is significantly reduced in serum from COPD patients and smokers when compared to non-smokers (134), which could further explain the increased level of adenosine in COPD patients.

## Therapeutic Targeting of Adenosine

### Targeting Hypoxia Signaling

Direct therapeutic targeting of the hypoxia signaling pathway could profoundly modulate the adenosine signaling pathway. Pharmacologic compounds have been developed for normoxic stabilization of HIFs by functioning as inhibitors of PHDs. Several preclinical studies show that these compounds can be given to animals that are kept under normoxic conditions, and result in robust stabilization of HIFs (170, 171). In line with this concept, preclinical studies have shown that pretreatment with the HIF activator dimethyloxalylglycine (DMOG) is associated with attenuated organ injury in the heart, lungs, or kidneys (72, 172, 173). Moreover, recently, several pharmaceutical companies have developed HIF activators as orally available compounds and several ongoing clinical trials have used them in patients for the treatment of anemia associated with chronic kidney disease. These compounds include roxadustat (FG-4592, sponsored by FibroGen, Astellas, & AstraZeneca), vadadustat (AKB-6548, sponsored by Akebia), and daprodustat (GSK-1278863, sponsored by GlaxoSmithKline). Based on phase 3 clinical trials showing efficiency in increasing hemoglobin levels in patients with anemia associated with renal insufficiency (174, 175), roxadustat has been approved for treating chronic kidney disease-related anemia in China and is currently in phase 3 clinical trials in the United States. In the meantime, several phase 2 clinical trials indicated that oral vadadustat is safe and effective as a treatment for anemia in patients with non-dialysis-dependent chronic kidney diseases (176, 177), and in patients receiving hemodialysis previously received erythropoiesis-stimulating agents (178). Currently, vadadustat is evaluated by a randomized, double-blinded and placebo-controlled phase 2 clinical trial as a treatment of COVID-19 associated ARDS (**Table 1**) (180). These oral available HIF activators could potentially be efficient for enhancing adenosine signaling pathways in patients for the prevention of acute lung injury. On the other hand, HIF inhibitors could potentially inhibit adenosine signaling as a therapeutic approach for chronic lung diseases. Currently, HIF-2α inhibitors such as PT2385 and PT2977 have been investigated by clinical trials mainly as novel therapeutic approaches for renal cell carcinoma (181). However, HIF-1α specific inhibitor has yet to be developed for clinical use, which will be crucial for the inhibition of adenosine signaling in chronic lung diseases.

**Table 1:** Clinical trials targeting adenosine signaling in lung diseases.

Drug(Company)	Target	Status	Target disease	Clinical trial gov identifier	References
**GW328267X**	A_2A_ adenosine receptor agonist	Completed Phase 1	Acute lung injury	**** *NCT01640990*	
**PBF-680**	A1 adenosine receptor antagonist	Completed Phase 2	Asthma	*NCT01939587* ****	
**PBF-680**	A1 adenosine receptor antagonist	Recruiting Phase 2	Asthma	*NCT02635945*	
**PBF-680**	A1 adenosine receptor antagonist	Completed Phase 1	Asthma	*NCT01845181*	
**PBF-680**	A1 adenosine receptor antagonist	Completed Phase 1	Asthma	*NCT02208973*	
**PBF-680**	A1 adenosine receptor antagonist	Recruiting Phase 2	Persistent, mild-to-moderate atopic asthma	*NCT03774290*	
**Regadenoson**	A_2A_ adenosine receptor agonist	Completed Phase4	As stress agents for myocardial perfusion imaging in asthma or COPD patients	*NCT00862641*	(179)
**Dipyridamole**	Equilibrative nucleoside transporter inhibitor	Recruiting Phase 2	COVID-19; SARS-CoV-2 infection	*NCT04391179*	
**Dipyridamole**	Equilibrative nucleoside transporter inhibitor	Recruiting Phase 2	COVID-19 pneumonia; Vascular complications	*NCT04424901*	
**Vadadustat**	Hypoxia-inducible factor prolyl hydroxylase (HIF-PH) inhibitor	Recruiting Phase 2	Acute respiratory distress syndrome; coronavirus infection	*NCT04478071*	

### Targeting Adenosine Receptors

Adenosine signaling could potentially be targeted for lung protection during acute lung inflammation *via* direct administration of adenosine or utilizing specific adenosine receptor agonists in both preclinical and clinical settings (182, 183). Several preclinical studies have indicated that direct administration of adenosine attenuates lung injury (184, 185). The safety of adenosine administration has also been supported by previous clinical studies (186, 187). However, due to the short half-life of adenosine *in vivo*, adenosine analogs might be a more feasible option. Adenosine receptor agonists have been developed for preclinical and clinical use (188). For example, pretreatment of A2AAR agonist ATL202 inhibits LPS-induced PMN recruitment, reduced the release of inflammatory cytokines in the lung, and reduced vascular leakage in mice (144). A2AAR agonist GW328267C improves lung function in three models of ALI (HCl instillation 1 h, LPS instillation 16 h, and live *Escherichia coli* instillation) in rats (189). The delivery of A2BAR-specific agonist BAY 60-6583 attenuate pulmonary edema, inhibits lung inflammation, and improves histologic lung injury in murine ALI (73, 107). Furthermore, mice treated with BAY60-6583 show attenuated oleic acid (OA)-induced ALI by inhibiting alveolar epithelial cell apoptosis (190). However, only A1AR, A2AAR, and A3AR agonists have been evaluated in the clinical setting while the safety and efficacy ofA2BAR agonists have yet to be established by clinical studies (188). The usage of adenosine receptor agonists in clinical trials related to lung injury is summarized in **Table 1**.

Adenosine receptor antagonists have been developed as treatment of chronic lung diseases in both preclinical and clinical settings. For instance, LASSBio-897 (3-thienylidene-3, 4-methylenedioxybenzoylhydrazide) can block the activity of A2AAR agonist and has anti-inflammatory and anti-fibrotic role in a mouse model of silicosis (191). Additionally, the treatment of A2BAR antagonist CVT-6883 dampens lung inflammation, reduces fibrosis, and attenuates alveolar airspace enlargement in ADA-deficient mice (192). Similarly, CVT-6883 treatment reduced inflammation and lung fibrosis in murine bleomycin-induced lung injury (192). Finally, A1AR antagonist PBF-680 has been and is currently being evaluated by several phase 1 and phase 2 clinical trials as a treatment of asthma (**Table 1**). Although adenosine receptor antagonists have been investigated for inflammatory conditions, neurodegenerative diseases, and mood disorders (193), their potential impact on chronic lung diseases needs to be further evaluated.

### Targeting Adenosine Metabolism

Adenosine signaling could also be targeted *via* modification of adenosine metabolism. For instance, inhibition or deletion of ENT1/2 elevates extracellular adenosine levels in lung tissue and improves pulmonary function by activating A2AAR and A2BAR receptor and preventing NLRP3 inflammasome activation in *Pseudomonas aeruginosa* infection-induced acute lung injury in mice (194). Moreover, ENT inhibitor dipyridamole treatment decreases adenosine uptake, and in turn improves vascular barrier and reduces neutrophil accumulation in acute pulmonary inflammation in preclinical studies (108, 123, 195). Along the same line, dipyridamole is currently investigated by several clinical trials as a treatment for COVID-19 and associated vascular manifestation (NCT04391179, NCT04424901, **Table 1**). Besides targeting ENTs, ADA administration reduced lung pathology in IL-13 transgenic mice, which spontaneously develop lung inflammation, alveolar destruction, and fibrosis (196). Furthermore, PEGylated adenosine deaminase is currently employed as an enzyme replacement therapy for patients suffering adenosine deaminase severe combined immunodeficiency (197) and has lately been shown to alleviate fibrosis and inflammation in a murine model of systemic sclerosis (198). PEGylated adenosine deaminase should be further investigated as a therapeutic approach for chronic lung diseases.

## Conclusion

Adenosine signaling is one of the most crucial mediators in the cross-talk between hypoxia and inflammation. In this review, many studies suggest that targeting hypoxia and adenosine signaling could be a promising therapeutic approach for ARDS and chronic lung diseases. However, further investigation is needed to address the knowledge gaps in the mechanism of how HIF-adenosine contributes to different disease conditions and how to target this pathway in patients. For instance, the functional link between HIF and adenosine pathway in viral pneumonia induced ARDS needs to be established, especially for COVID-19 associated ARDS. Furthermore, the functional role of the HIF-adenosine pathway needs to be demonstrated in COPD and asthma for the development of novel therapies targeting this pathway. Pharmacological agents to modulate adenosine signalings, such as adenosine receptor antagonists and PEGylated adenosine deaminase, have been investigated in several disease conditions. However, its potential use for chronic lung diseases needs to be further evaluated. Taken together, a detailed understanding of the functional role of the HIF-adenosine axis is needed for the development of efficient and safe therapy in pulmonary diseases.

## Author Contributions

XL drafted the manuscript. NB assisted with the literature search. TM revised the manuscript. KZ assisted with the figures. HE revised the manuscript and provided critical advice on the structure and content of the manuscript. XY drafted and finalized the manuscript and provided critical advice on the structure and content of the manuscript. All authors contributed to the article and approved the submitted version.

## Funding

This study was supported by R01 DK097075, POI-HL114457, R01-HL109233, R01-DK109574, R01-HL119837, R01-DK082509, R01-HL154720, and R01-HL133900 to HE, the American Thoracic Society Unrestricted Grant, American Heart Association Career Development Award (19CDA34660279), American Lung Association Catalyst Award (CA-622265), the Center for Clinical and Translational Sciences, McGovern Medical School Pilot Award (1UL1TR003167–01), the Parker B. Francis Fellowship to XY, and the China Scholarship Council State Scholarship Fund to XL.

## Conflict of Interest

The authors declare that the research was conducted in the absence of any commercial or financial relationships that could be construed as a potential conflict of interest.
